# Synthesis and Characterization of pH and Thermo Dual-Responsive Hydrogels with a Semi-IPN Structure Based on *N*-Isopropylacrylamide and Itaconamic Acid

**DOI:** 10.3390/ma11050696

**Published:** 2018-04-28

**Authors:** Syang-Peng Rwei, Huynh Nguyen Anh Tuan, Whe-Yi Chiang, Tun-Fun Way

**Affiliations:** Institute of Organic and Polymeric Materials, Research and Development Center for Smart Textile Technology, National Taipei University of Technology, #1, Sec 3, Chung-Hsiao E. Rd, Taipei, Taiwan; tuanhna@hcmute.edu.vn (T.H.N.A.); wheyichiang@gmail.com (W.-Y.C.); tfway1951@gmail.com (T.-F.W.)

**Keywords:** free radical polymerization, *N*-isopropylacrylamide, itaconamic acid, poly(NIPAM-co-IAM), pNIPAM, semi-IPN hydrogel, pH-responsive, thermo-responsive, swelling ratio

## Abstract

A series of semi-interpenetrating polymer network (semi-IPN) hydrogels were synthesized and investigated in this study. Linear copolymer poly(*N*-isopropylacrylamide-co-itaconamic acid) p(NIPAM-co-IAM), which is formed by copolymerization of *N*-isopropylacrylamide (NIPAM) and itaconamic acid (IAM, 4-amino-2-ethylene-4-oxobutanoic acid), was introduced into a solution of NIPAM to form a series of pH and thermo dual-responsive p(NIPAM-co-IAM)/pNIPAM semi-IPN hydrogels by free radical polymerization. The structural, morphological, chemical, and physical properties of the linear copolymer and semi-IPN hydrogels were investigated. The semi-IPN hydrogel showed high thermal stability according to thermal gravimetric analyzer (TGA). Scanning electronic microscopy (SEM) images showed that the pore size was in the range of 119~297 µm and could be controlled by the addition ratio of the linear copolymer in the semi-IPN structure. The addition of linear copolymer increased the fracture strain from 57.5 ± 2.9% to 91.1 ± 4.9% depending on the added amount, while the compressive modulus decreased as the addition increased. Moreover, the pH and thermo dual-responsive properties were investigated using differential scanning calorimetry (DSC) and monitoring the swelling behavior of the hydrogels. In deionized (DI) water, the equilibrium swelling ratio of the hydrogels decreased as the temperature increased from 20 °C to 50 °C, while it varied in various pH buffer solutions. In addition, the swelling and deswelling rates of the hydrogels also significantly increased. The results indicate that the novel pH-thermo dual-responsive semi-IPN hydrogels were synthesized successfully and may be a potential material for biomedical, drug delivery, or absorption application.

## 1. Introduction

Hydrogel is a type of polymer material with a three-dimensional network capable of absorbing water without dissolving. It can be molded into any form, shape, or size for many potential applications in many fields. In recent times, smart hydrogel has been studied extensively due to its phase transitions in response to external stimulus, including temperature, pH, ionic strength, light, pressure, redox, and electric field [1,2,3,4,5,6,7,8,9,10]. The typical smart hydrogel is thermal-responsive hydrogel, which has phase transitions in response to temperature changes, and poly(*N*-isopropylacrylamide) (pNIPAM) is the most widely investigated.

pNIPAM exhibits a lower critical solution temperature (LCST), about 32 °C in pure water, which is close to human body temperature [11,12,13]. At temperatures below its LCST, the cross-linked pNIPAM is hydrophilic and absorbs water to a swollen state. On the contrary, it is hydrophobic and water is removed from the network to a shrunken state with decreased volume material [13,14]. The hydration and dehydration of the cross-linked pNIPAM may be explained by the inter- and intramolecular interaction within the polymer network [15,16]. Below LCST, it possesses strong hydrogen bonds between pNIPAM and water, causing the swollen state, while above LCST, the interaction with pNIPAM is stronger than the hydrogen bonds, leading to the shrunken state. Having this special feature, pNIPAM hydrogel can be used as a thermo-responsive carrier in many fields, such as in drug release [17,18], for bioactive molecule separation [19], as a catalyst [20], for cell harvesting [21], as a seal-healing hydrogel [22], etc. However, conventional pNIPAM hydrogels also have some disadvantages: they are brittle at room temperature and have poor mechanical properties, a low swelling ratio, and a slow response rate [23,24,25].

For several potential applications, such as drug delivery and absorbing systems, a pH and thermo dual-responsive hydrogel with a high swelling ratio, a fast response rate, and good mechanical properties is needed, because temperature and pH are important environmental factors in these systems. One of the most useful methods to meet these requirements is to prepare semi-interpenetrating (semi-IPN) hydrogels by introducing a high hydrophilic linear polymer into the pNIPAM network. Semi-IPN hydrogels are usually synthesized by simultaneous polymerization of a monomer system with a cross-linking agent in the presence of natural polymer [18,25,26,27] or synthetic linear polymer chains [24], which will be physically entangled within the polymer network. The mechanical properties and response rate of the obtained hydrogels can thereby be improved. Moreover, to obtain a high hydrophilic pH-responsive polymer that has one or more properties responding to pH change, a carboxylic acid monomer such as acrylic acid, itaconic acid, or methacrylic acid may be introduced to function as a pH-sensitive segment [13,28,29]. Therefore, the obtained polymers are not only thermo- but also pH-sensitive.

In our previous studies, itaconamic acid (IAM) was used as a pH-sensitive segment to prepare thermo- and pH-responsive copolymers [30,31,32]. In addition, we reported the synthesis of conventional hydrogel between NIPAM, IAM, and β-cyclodextrin with high swelling ratio, because IAM has two hydrophilic functional groups simultaneously (–NH_2_ and –COOH) [33]. In the present work, unlike the conventional structure, the semi-IPN hydrogels are investigated. The pH-thermo dual-responsive p(NIPAM-co-IAM)/pNIPAM semi-IPN hydrogels (referred to as PNA hydrogels) were prepared by free radical polymerization of NIPAM in the presence of the linear copolymer p(NIPAM-co-IAM) and the cross-linker. The linear copolymer was expected to entangle physically in the polymer network. To the best of our knowledge, this is the first time NIPAM and IAM have been combined in a semi-IPN hydrogel. The chemical structures of the linear copolymer and hydrogels were investigated by FTIR and ^1^H NMR. The molecular weight and hydrodynamic radius of linear copolymer were measured using gel permeation chromatography (GPC) and dynamic light scattering (DLS), respectively. The other properties were further characterized using thermal gravimetric analyzer (TGA), dynamic mechanical analyzer (DMA), SEM and rheological measurement. Finally, the LCSTs and swelling behavior of hydrogels were investigated in various pH buffer solutions (PBS) and temperatures. It is expected that these PNA hydrogels will have outstanding performance in a drug delivery or absorption application.

## 2. Materials and Methods

### 2.1. Materials

*N*-isopropylacrylamide (NIPAM; C_6_H_11_NO) obtained from Aldrich Chemical Corp (Saint Louis, MO, USA). was purified through recrystallization from n-hexane twice. Itaconamic acid (IAM; C_5_H_7_NO_3_) was prepared according to the method disclosed in US patent publication No. 2013/0172490 [34]. Ammonium persulfate (APS; (NH_4_)_2_S_2_O_8_) as an initiator was purchased from Aencore Chemical Pty. Ltd. (Surrey Hills, Australia) and used as received. *N*,*N*,*N*′,*N*′-tetramethylethylenediamine (TEMED; C_6_H_16_N_2_) as a catalyst and *N*,*N*′-methylenebisacrylamide (MBA; C_7_H_10_N_2_O_2_) as a cross-linker were purchased from Alfa Aesar Co. (Tewksbury, MA, USA) and used as received without any further purification. *N*,*N*-Dimethylformamide (DMF) was obtained from Macron Fine Chemicals. The buffer solution of pH 4, pH 7, and pH 10 was obtained from J. T. Baker Chemical Corp. (Center Valley, PA, USA), the buffer solution of pH 5 was obtained from Alfa Aesar Co., and buffer solution of pH 6 and pH 8 was obtained from Sigma Aldrich (Saint Louis, MO, USA).

### 2.2. Preparation of Linear Copolymer p(NIPAM-co-IAM)

**Figure 1:**
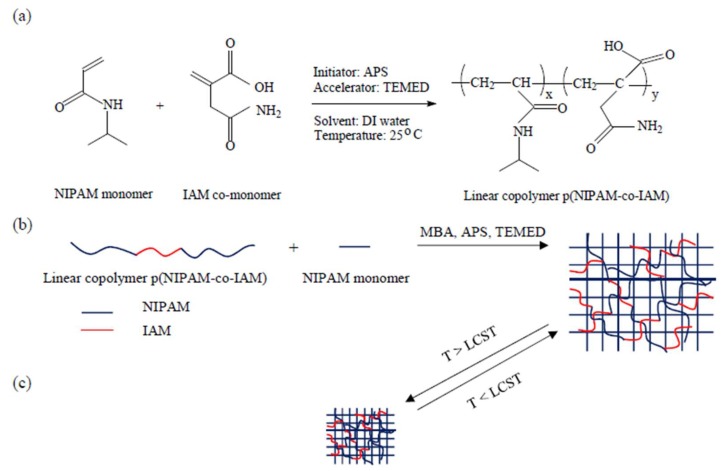
Preparation scheme of (**a**) Linear copolymer poly(*N*-isopropylacrylamide-co-itaconamic acid) (p(NIPAM-co-IAM)); (**b**) Semi-interpenetrating polymer network (semi-IPN) hydrogels; and (**c**) The coil-to-globule transition of semi-IPN hydrogels below and above lower critical solution temperature (LCST). APS, ammonium persulfate; TEMED, *N*,*N*,*N*′,*N*′-tetramethylethylenediamine; DI, deionized; MBA, *N*,*N*′-methylenebisacrylamide.

The preparation of linear copolymer p(NIPAM-co-IAM) is shown in Figure 1a. It was prepared by free radical polymerization, employing APS as an initiator. First, 1.5 g of NIPAM and 0.13 g of IAM were dissolved together in 6.0 mL of deionized (DI) water by continuous stirring in a flask under a nitrogen atmosphere and placed in an ice-water bath. Then, 3.5 mL aqueous solution of APS (0.4792 g APS/25 mL DI water) and 7.0 mL aqueous solution of TEMED (4 mL TEMED/25 mL DI water) were added to initiate the polymerization. After polymerization in the ice-water bath for 4 h, the solution was ready for the subsequent processes. At this point, 2 mL of solution was dialyzed against DI water using a dialysis membrane with a molecular weight cutoff of 10,000 g/mole for 1 week (refreshed every half-day). Finally, the obtained solution was freeze-dried at −50 °C to completely remove water for further investigation. The linear homopolymer pNIPAM was prepared by a similar method as described above for comparison.

### 2.3. Preparation of Semi-IPN Hydrogels

The preparation of semi-IPN hydrogels is shown in Figure 1b. They were prepared by mixing linear copolymer p(NIPAM-co-IAM) solutions (≈100 mg/mL) and NIPAM solutions (100 mg/mL, containing 3.0 mole % MBA) in a vial with various designated ratios (0:10, 1:9, 2:8, 3:7, and 4:6, *v*/*v*) in an ice-water bath. The mixture was purged with nitrogen for 30 min until the solution became homogeneous. After that, TEMED (5% mole) and APS (2% mole) solution were added as a redox initiator pair to initiate the final reaction mixture. The solution was mixed thoroughly and then quickly poured into a cylindrical glass mold, sealed immediately, and kept at room temperature for 24 h. The ratio 0:10 represents the conventional pNIPAM hydrogel. The obtained hydrogels were carefully removed from the mold and immersed in DI water, which was refreshed every half-day for 1 week to eliminate the unreacted reagents. Finally, they were freeze-dried at −50 °C to completely remove water for further investigation. The feed ratios are given in Table 1.

### 2.4. Characterization

#### 2.4.1. FITR Measurement

Fourier transform infrared (FTIR) spectra were measured by a Perkin Elmer Spectrum RXI FTIR instrument (Waltham, MA, USA) within 4000–400 cm^−1^ with scan resolution of 4.00 cm^−1^. Attenuated total reflectance was applied to NIPAM and IAM monomer, the dried conventional pNIPAM, and semi-IPN hydrogels. Background measurements were performed and subtracted for all the samples.

#### 2.4.2. ^1^H NMR Measurement

Proton nuclear magnetic resonance (^1^H NMR) spectra of the monomers, dried linear copolymer p(NIPAM-co-IAM), and homopolymer pNIPAM were measured using a Bruker Advance 300MHz NMR spectrometer (Billerica, MA, USA) by weighing 10 mg of a test sample and dissolving in 1 mL of DMSO-d6 placed in a standard 507-HP NMR test tube.

#### 2.4.3. Gel Permeation Chromatography (GPC)

A Viscotek GPC system from Malvern Ltd. (Malvern, UK) was used to measure the weight average molecular weight (Mw), number average molecular weight (Mn), and polydispersity index (PDI; Mw/Mn) of the linear copolymer p(NIPAM-co-IAM) and homopolymer pNIPAM. The test sample was prepared by weighing about 15 mg of test material and dissolving it in 10 mL of DMF under the following conditions: column 300 × 8 mm, flow rate of 1 mL/min, temperature of the column set to 60 °C, temperature of the detector set to 60 °C, and injection quantity of test sample and standard was 150 μL each.

#### 2.4.4. Dynamic Light Scattering

Hydrodynamic radii of linear copolymer p(NIPAM-co-IAM) and homopolymer pNIPAM were measured by dynamic light scattering (DLS; Malvern 1000 HSA, Malvern, UK). The 0.5 wt % aqueous solutions of samples were measured at 25 °C and 40 °C.

#### 2.4.5. Differential Scanning Calorimetry (DSC)

The lower critical solution temperature (LCST) of the hydrogels was measured using a differential scanning calorimeter (DSC 8000, Perkin Elmer, Waltham, MA, USA). The dried hydrogel samples were immersed in DI water and pH buffer solution with different pH values for about 5 days at room temperature. The swelling hydrogel samples were moved out, excess water was wiped off with filter paper, and they were tested using DSC at the heating rate of 1 °C/min in the temperature range of 20–50 °C. The LCSTs of the hydrogel samples were determined at the maximum endothermic point.

#### 2.4.6. Thermal Stability

The thermal stability of the hydrogels was investigated using a thermal gravimetric analyzer (TGA) (TG 209 F3, Netzsch, Selb, Germany). The dried samples of linear copolymer p(NIPAM-co-IAM), conventional pNIPAM, and semi-IPN hydrogel were heated from 35 °C to 600 °C under a nitrogen atmosphere at a heating rate of 10 °C/min.

#### 2.4.7. Scanning Electron Microscopy (SEM) Analysis

Surface morphology and cross-sections of the dried hydrogels were observed using a Hitachi S-4700 scanning electron microscope (SEM) (Hitachi, Tokyo, Japan). The hydrogels were swollen to equilibrium in DI water at 20 °C and then freeze-dried at −50 °C to completely remove water. The freeze-dried samples were sputter-coated with gold for 10 min to enhance conductivity for better observation. SEM images were acquired at an accelerating voltage of 15 kV.

#### 2.4.8. Rheological Measurement

Oscillatory parallel-plate rheological measurements were performed using an Anton Paar MCR 301 rheometer (Anton Paar, Graz, Austria). The storage modulus (G’) and loss modulus (G’’) were measured as a function of time in the range of 10–500 s with a frequency of 1 Hz, as a function of frequency in the range of 0.1–10 Hz at room temperature. Moreover, G’ and G’’ were also measured as a function of temperature in the range of 25–50 °C with a frequency of 1 Hz to investigate the influence of temperature on the rheological properties of materials. All tests were performed at a strain of 1%.

#### 2.4.9. Mechanical Properties

The mechanical properties of the hydrogels were tested using a dynamic mechanical analyzer (DMA) (DMA 7e, Perkin Elmer, Waltham, MA, USA) at room temperature. The compression tests were performed on the cylindrical hydrogel samples (Φ10 mm × 7 mm) at a compression rate of 200 mN/min until cracking. The compressive modulus was confirmed by the slope of the stress-strain curve in the strain range of 10–30%. Fracture strain and stress were determined from the point where the stress value started dropping. All tests were performed three times.

#### 2.4.10. Swelling Behavior Measurements

To investigate the pH-thermo dual-responsive properties of the hydrogels, the equilibrium swelling ratio (SR) as a function of temperature and pH value was studied gravimetrically. The freeze-dried hydrogels were immersed in DI water at different temperatures in the range of 20–50 °C and in buffer solution with pH values ranging from 4 to 10 at 25 °C. After 5 days, the equilibrium swollen hydrogels were wiped off the surface water with filter paper. The SR was confirmed according to the following equation:

(1) $$\mathrm{SR}=\frac{W_{s}-W_{d}}{W_{d}}$$

where W_s_ is the weight of the equilibrium swollen hydrogel and W_d_ is the weight of the freeze-dried hydrogel.

#### 2.4.11. Swelling Kinetics Measurements

To investigate the swelling kinetics of the hydrogels, the freeze-dried samples were immersed in DI water at 20 °C. At predetermined time points, the swollen hydrogels were removed, water was wiped off the surface by filter paper, and they were weighed by an electronic balance. The SR was also calculated using the equation above.

#### 2.4.12. Deswelling Kinetics Measurements

To investigate the deswelling kinetics of the hydrogels, the freeze-dried samples were first immersed in DI water at 20 °C. After 5 days, the equilibrium swollen hydrogels were quickly transferred to DI water at 45 °C. At predetermined time points, the shrunken hydrogels were removed, water was wiped off the surface by filter paper, and they were weighed by an electronic balance. Water retention (WR) was calculated according to the following equation:

(2) $$\mathrm{WR}\left(\text{\%}\right)=\frac{W_{t}-W_{d}}{W_{e}-W_{d}}\times \;100$$

where W_t_ is the weight of the shrunken hydrogel at specific time t at 45 °C, W_d_ is the weight of the freeze-dried hydrogel, and W_e_ is the weight of the equilibrium swollen hydrogel at 25 °C.

## 3. Results and Discussion

### 3.1. Preparation of Semi-IPN Hydrogels

The preparation of semi-IPN hydrogels was performed in two steps. In the first step, linear copolymer p(NIPAM-co-IAM) was synthesized by free radical copolymerization of NIPAM and IAM using APS as an initiator and TEMED as a catalyst. In the second step, NIPAM monomer, linear copolymer p(NIPAM-co-IAM), and MBA as a cross-linker were mixed together in DI water in the designed ratios. Then, APS and TEMED were added to the solution in an ice bath. In both cases, APS was added to produce sulfate-free radicals to initiate the polymerization reaction and TEMED was added to accelerate the process by facilitating homolytic scission on APS moieties [35]. Finally, the pNIPAM network was formed in the presence of the linear copolymer and a semi-IPN hydrogel was obtained.

**Figure 2:**
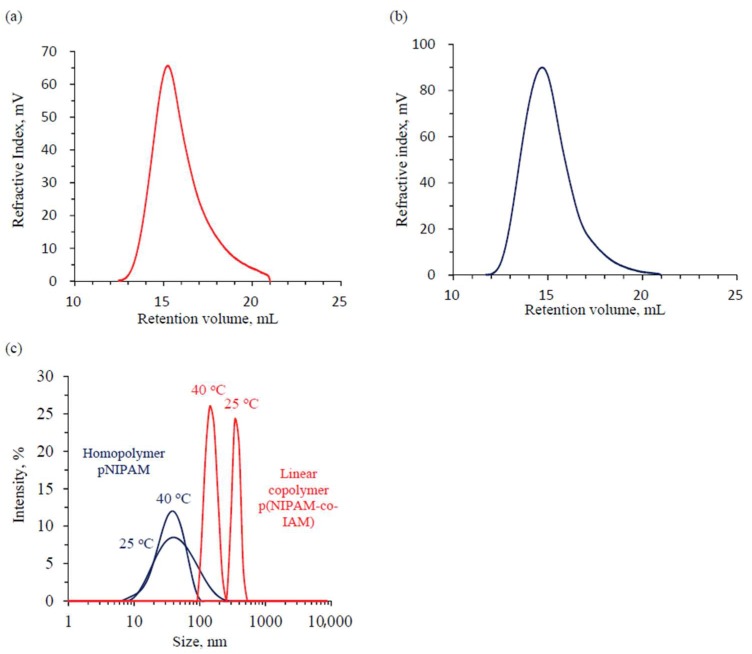
(**a**) Gel permeation chromatography (GPC) trace of linear copolymer p(NIPAM-co-IAM) and (**b**) Homopolymer pNIPAM; (**c**) Dynamic light scattering (DLS) of linear copolymer p(NIPAM-co-IAM) and homopolymer pNIPAM.

The GPC curves of the linear copolymer p(NIPAM-co-IAM) and homopolymer pNIPAM are shown in Figure 2a,b, respectively. The number average molecular weight (Mn) and polydispersity index (PDI) of the linear polymers are shown in Table 2. The PDI was rather high and not significantly affected by the fraction of IAM monomer. It was consistent with the report on the synthesis of linear copolymer poly(*N*-isopropylacrylamide-co-hydroxyethylmethacrylate) by Yan et al. [24]. The Mn of p(NIPAM-co-IAM) (5.26 × 10^4^ g/mol) was lower than pNIPAM (23.9 × 10^4^ g/mol). This may be from the influence of alpha-hydrogens positioned simultaneously at the carbonyl alpha and allyl alpha positions from IAM composition units. These functional groups could increase the activity of free radicals on the polymer chains. Then, these radicals would easily be combined with the radical originating from the initiator and the chain termination occurred quickly.

Hydrodynamic diameters (D_H_) of the linear polymers at 25 °C and 40 °C are shown in Figure 2c. The D_H_ of linear copolymer p(NIPAM-co-IAM) and homopolymer pNIPAM at 40 °C are 151.9 ± 20.3 and 38.4 ± 2.8 nm, respectively; at 25 °C the obtained values are 292.3 ± 38.3 and 51.2 ± 6.4 nm, respectively. The D_H_ of p(NIPAM-co-IAM) is higher than the pNIPAM, and this may be due to the influence of the IAM fraction within the linear copolymer. In DI water, the solution of p(NIPAM-co-IAM) is acidic, with pH 3.8 (measured by pH meter), since some amino groups from IAM segments were ionized and represented as a –NH_3_^+^ form. The copolymer chains with positive charges had stronger repulsion force, leading to increased D_H_ compared to the homopolymer. Moreover, as the temperature increased from 25 °C (below LCST) to 40 °C (above LCST), the D_H_ of polymer/copolymer decreased dramatically because of coil-to-globule transition behavior [36].

On the other hand, the linear copolymer p(NIPAM-co-IAM) had a larger hydrodynamic diameter and lower Mn than linear pNIPAM. The result is not consistent with the reports of Liu et al. and Yin et al. for poly(dimethylsiloxane) and hydrolyzed polyacrylamide, respectively [37,38]. This may be also due to the influence of ionized IAM fraction, as discussed above.

### 3.2. ^1^H NMR Measurement

**Figure 3:**
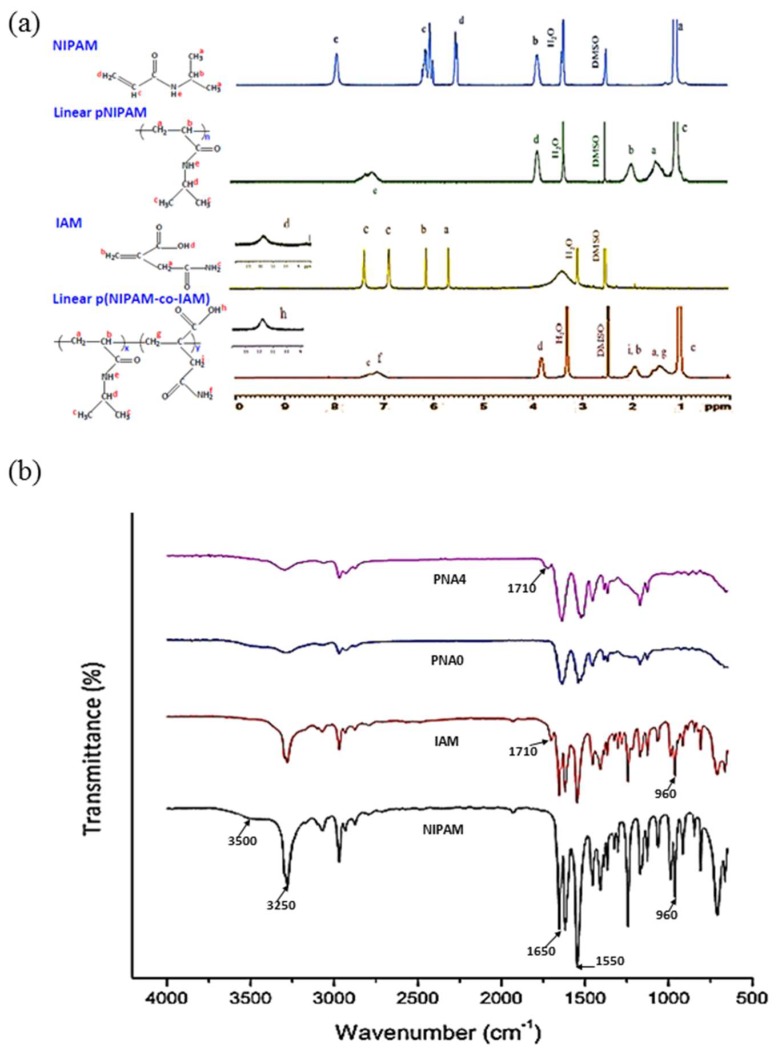
(**a**) ^1^H NMR and (**b**) FTIR spectra of monomers, linear polymers, and semi-IPN hydrogels.

Figure 3a shows ^1^H NMR spectra of NIPAM monomer, IAM co-monomer, linear homopolymer pNIPAM, and linear copolymer p(NIPAM-co-IAM). The characteristic peaks from IAM are at δ = 5.7 ppm (–CH_2_–), δ = 6.2 ppm (CH_2_=), and δ = 6.9–7.4 (–CO–NH_2_). The characteristic peaks from NIPAM are at δ = 0.8–1.2 ppm (CH_3_–, isopropyl), δ = 3.8–4.1 ppm (CH, isopropyl), δ = 5.6 and 6.1 ppm (CH_2_=CH–), and δ = 7.9 (–NH–, amide). From the ^1^H NMR spectra of linear pNIPAM and linear p(NIPAM-co-IAM) in Figure 3a, bands a and b are attributed to the –CH_2_–CH– group from the polymer chain, band c is attributed to the isopropyl group, band d is attributed to the CH–NH– group, and bands e and f are attributed to the NH_2_– group from the IAM and NH group from NIPAM. The peaks from NIPAM at δ = 5.6 and 6.1 ppm (bands c and d) and from IAM at δ = 5.7 and 6.1 ppm (bands a and b) are absent in the spectra of linear pNIPAM and linear p(NIPAM-co-IAM), indicating successful synthesis of linear pNIPAM and linear p(NIPAM-co-IAM).

The molar ratio of NIPAM/IAM in the linear p(NIPAM-co-IAM) was determined by ^1^H NMR, where the area of the peak at δ = 3.7–4.0 ppm is defined as 1 [30]. Integration of the area under the peak at δ = 1.2–1.7 ppm (2H from NIPAM and 2H from IAM) and δ = 1.7–2.2 ppm (1H from NIPAM and 2H from IAM) confirmed the presence of copolymerized p(NIPAM-co-IAM) with quantitative conversion of the molar ratio of NIPAM/IAM = 100/10.

### 3.3. FTIR Measurement

The FTIR spectra of NIPAM monomer, IAM co-monomer, conventional pNIPAM, and semi-IPN hydrogels are shown in Figure 3b. In the spectrum of NIPAM monomer, the amide C=O stretching peak is shown at about 1650 cm^−1^, the amine N–H bending peak is shown at about 1550 cm^−1^, the amine N–H stretching peak is shown at 3500~3250 cm^−1^, and the mono-substituted C=C peak is shown at 960 cm^−1^ [39]. In the spectrum of IAM monomer, the C=C peak is also shown at 960 cm^−1^ and has a distinct characteristic peak at 1710 cm^−1^, which corresponds to the C=O peak in the carboxylic group [30]. After polymerization, the spectra of conventional pNIPAM and semi-IPN hydrogels showed an absence of the peak at about 960 cm^−1^, indicating that C=C is formed into C–C in the three-dimensional hydrogels.

As shown in Figure 3b, the spectrum of semi-IPN hydrogel showed a peak at 1710 cm^−1^ with the intensity lower than the peak in the spectrum of IAM monomer. Thus, the FTIR spectra confirmed successful synthesis of linear copolymer p(NIPAM-co-IAM). Moreover, the FTIR spectra also confirmed successful synthesis of the conventional pNIPAM and semi-IPN hydrogels.

### 3.4. Thermal Gravimetric Analyses

**Figure 4:**
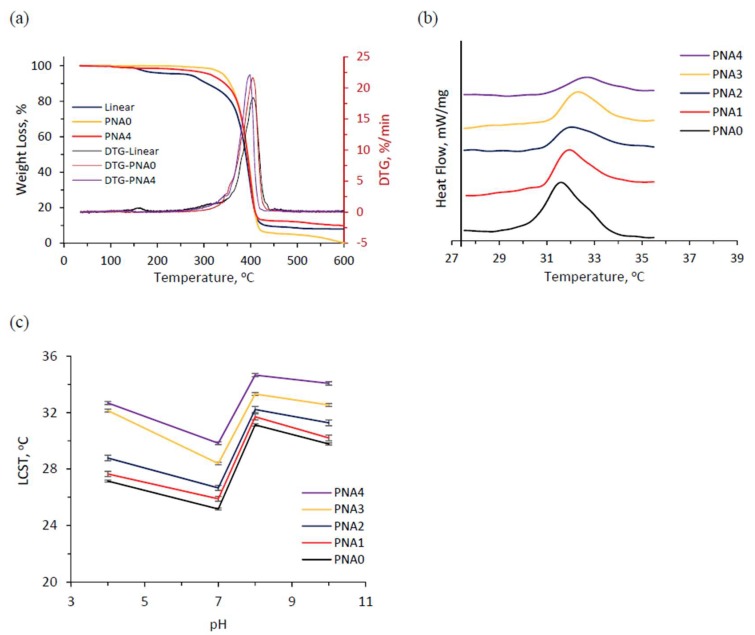
(**a**) TGA and DTG curves of linear copolymer p(NIPAM-co-IAM), conventional pNIPAM, and semi-IPN hydrogels; (**b**) DSC traces of hydrogels in DI water; (**c**) LCSTs of equilibrium swollen hydrogels in PBS with pH values ranging from 4 to 10 at 25 °C.

The thermal stability of the hydrogels was tested by thermogravimetric analysis (TGA). Figure 4a shows the thermal degradation curves of linear copolymer p(NIPAM-co-IAM), conventional pNIPAM, and semi-IPN hydrogels. For p(NIPAM-co-IAM), the thermal degradation included two stages. The first stage showed 4% weight loss at around 180 °C, while the second stage showed that weight loss decreased rapidly from 270 to 420 °C and remained about 8% at 600 °C. This result could be explained by the degradation of IAM segments and the rest of these groups linked to NIPAM. The same result was reported by Sousa et al. for poly(*N*-isopropylacrylamide)-co-acrylamide gels [40]. For conventional pNIPAM hydrogel, the thermal degradation took place quickly from 320 to 420 °C and decreased slowly until it was completely decomposed, which was attributed to the decomposition of the pNIPAM hydrogel network. For semi-IPN hydrogel, we found about 1.5% weight loss at around 180 °C relating to the degradation of linear copolymer. The second stage happened at about 300 °C, which was between the p(NIPAM-co-IAM) and pNIPAM hydrogel, showing a strong interaction between the linear copolymer and hydrogel network [41]. The TGA curves also proved that semi-IPN hydrogel has good thermal stability, with a residual mass of 10% at 600 °C, higher than the conventional pNIPAM hydrogel, with 0.1% at the same temperature. In comparison with other semi-IPNs based on pNIPAM, Li et al. reported that the maximum residual mass of thermal-responsive hydrogels poly[(*N*-isopropylacrylamide)-co-(aminoethyl methacrylate β-cyclodextrin)] was 10% [17]. On the other hand, Wei et al. reported that a maximum residual mass of 13% was found in salecan/pNIPAM semi-IPN hydrogel [27]. The difference in thermal stability of semi-IPN hydrogels may be due to the molecular weight and the proportion of introduced linear polymer. Higher molecular weight and higher proportion of linear polymer led to higher thermal stability of semi-IPN hydrogel.

### 3.5. Differential Scanning Calorimetry (DSC)

The LCSTs of the hydrogels were characterized using DSC analysis. Figure 4b shows the DSC curves at the heating rate of 1 °C/min of the swelling PNA hydrogels with different molar fractions added to the linear copolymer p(NIPAM-co-IAM) that was introduced. The LCSTs of the hydrogels increased slightly in the range of 32.0–32.7 °C as the volume of added p(NIPAM-co-IAM) solution increased (i.e., the increase of IAM molar fraction), and the conventional pNIPAM hydrogel without IAM segment had the lowest LCST at 31.6 °C. The results can be explained by the contribution of the hydrophilic functional groups, such as carboxyl and amino groups, in the linear copolymer from IAM formed hydrogen bonds with water, leading to increased LCST. This phenomenon is consistent with our previous research, where poly(NIPAM-co-IAM) was synthesized in DMF solvent with 2,2′-azobis(isobutyronitrile) as the initiator [30]. The results also show that semi-IPN hydrogels are thermo-sensitive.

To investigate the pH sensitivity of the semi-IPN hydrogels, they were immersed in pH buffer solution at pH 4, pH 7, pH 8, and pH 10 until equilibrium swelling. The LCST data from DSC traces at the heating rate of 1 °C/min of equilibrium swollen hydrogels are shown in Figure 4c. The results show that the LCST of semi-IPN hydrogels is higher than that of conventional pNIPAM hydrogel. In the case of semi-IPN hydrogels, LCST increased as more linear copolymer p(NIPAM-co-IAM) was introduced into the network. This is due to the hydrogels being more hydrophilic with more molar fraction of IAM inside the network structure. The LCST trends of the hydrogels in various pH buffer solutions are shown in Figure 4c. In general, LCST decreased with increased pH value in the range of 4 to 7 and reached the lowest level at pH 7. Then, LCST strongly increased at pH 8. However, it slightly decreased again at pH 10. The results are in agreement with the report by Pei et al. for the homopolymer pNIPAM hydrogel but are different from the result of the copolymer p(NIPAM-co-AAc) hydrogel [13]. The reason is that the IAM monomer contains two types of functional groups, –COOH and –NH_2_, compared to AAc monomer, which contains only the –COOH group. In the weak acid (pH 4) and weak basic (pH 8) solution, probably the ionization took place at the –NH_2_ and –COOH functional groups, respectively. The –NH_3_^+^ and –COO^−^ ions could establish many hydrogen bonds between the polymer network and water to cause higher LCST. In the neutral pH 7 solution, ionization took place simultaneously in both functional groups, causing the ion concentration in the polymer network to rise sharply, leading to the salting out phenomenon and the hydrogen bonds being destroyed. Thus, the LCST at pH 7 was the lowest. In addition, under the effect of osmotic pressure as the ionic concentration in pH 10 solution increased, the water inside the polymer network ran away, resulting in destruction of hydrogen bonds. These results show the pH-responsive ability of semi-IPN hydrogels.

### 3.6. Morphology

**Figure 5:**
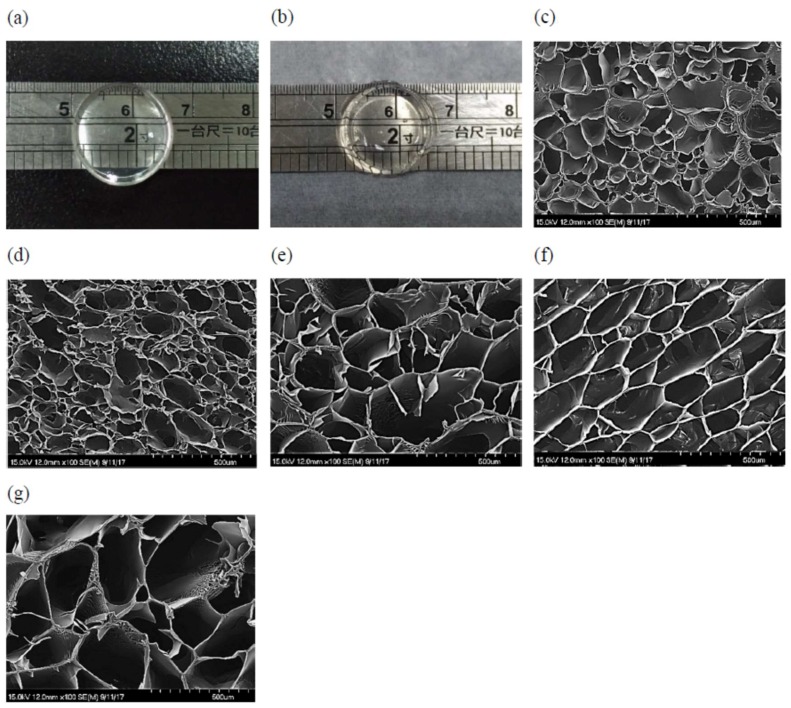
Optical images of (**a**) conventional pNIPAM and (**b**) semi-IPN hydrogels; and SEM images of conventional pNIPAM and semi-IPN hydrogels: (**c**) PNA0, (**d**) PNA1, (**e**) PNA2, (**f**) PNA3, and (**g**) PNA4.

Figure 5a,b show photographs of conventional pNIPAM and semi-IPN hydrogels, respectively. The transmittance of the two samples is quite similar, indicating that the linear copolymer p(NIPAM-co-IAM) did not alter the structural state of the semi-IPN hydrogel. SEM images of the hydrogels in Figure 5c–g shows a porous structure with a relatively uniform pore size distribution. Wei et al. reported that fewer closed pores (with small size) were observed in conventional pNIPAM hydrogel [27]. In another report, Liu et al. reported that conventional pNIPAM hydrogel showed the typical microporous morphology with dense walls [24]. In this study, conventional pNIPAM hydrogel showed a clear uniform hollow structure. Moreover, it was observed that the average pore size was significantly affected by the linear copolymer content introduced into the semi-IPN hydrogels. They are listed in Table 3. Specifically, the PNA1 sample had the smallest pore size, 119 ± 16 µm, and the PNA4 sample had the largest pore size, 297 ± 61 µm. This may be related to the hydrophilicity of IAM and cross-linking density of the samples. From PNA1 to PNA4 samples, the linear copolymer content increased, leading to an increase in hydrophilicity and a decrease in cross-linking density. It was reported that the lower cross-linking density produced a hydrogel with larger pore size [42]. Besides, the more hydrophilic hydrogels absorbed more water and produced larger space inside them. Generally, pore size can be controlled by changing the linear copolymer content introduced into the semi-IPN hydrogels.

### 3.7. Rheological Measurement

**Figure 6:**
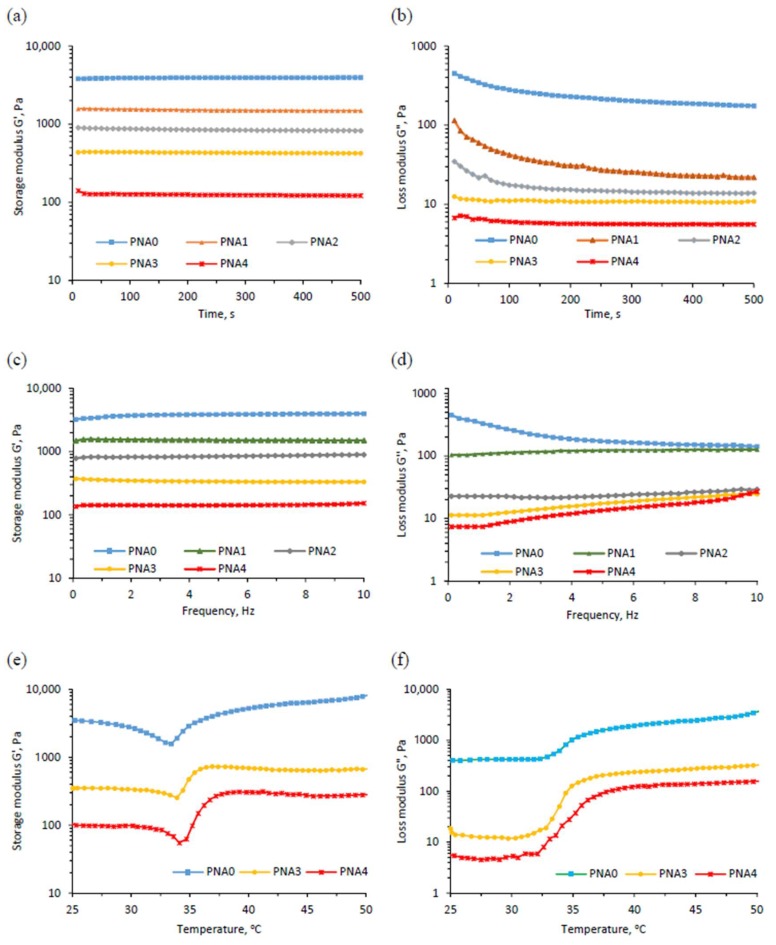
(**a**) Plots of storage modulus G’ as a function of time; (**b**) Plots of loss modulus G’’ as a function of time; (**c**) Plots of storage modulus G’ as a function of frequency; (**d**) Plots of loss modulus G’’ as a function of frequency; (**e**) Plots of storage modulus G’ as a function of temperature; (**f**) Plots of loss modulus G’’ as a function of temperature, for conventional pNIPAM and semi-IPN hydrogels.

The storage modulus (G’) and loss modulus (G’’) of conventional pNIPAM and semi-IPN hydrogels as a function of time and frequency are shown in Figure 6a–d. The results show that G’ values were independent of time and frequency and were much higher than G’’ values, showing a viscoelastic behavior of hydrogel materials [43]. It was also observed that G’ and G’’ values decreased with increased linear copolymer p(NIPAM-co-IAM) content introduced into the hydrogels. This could be explained by the flexibility of the polymer network. As the added volume of linear copolymer solution increased, the content of NIPAM and MBA decreased, leading to reduced cross-linking density in the obtained hydrogels. So, the flexibility of the hydrogels would increase due to generation of fewer entanglements in the hydrogel network to cause a reduction of elastic modulus [27,44].

The influence of temperature on G’ and G’’ values is shown in Figure 6e,f. It was observed that G’ and G’’ values decreased slightly as temperature was raised from 25 to 34 °C, while they increased rapidly at temperatures above 34 °C. The results confirm the thermal sensitivity of the hydrogels. Increasing temperature from 25 °C to 34 °C increased the thermal motion of the linear copolymer, leading to increased flexibility of the overall hydrogel network and reduced elastic modulus. As the temperature was raised above 34 °C, the interaction within the pNIPAM network was stronger than the hydrogen bonds between the pNIPAM and water [15]. Therefore, the absorbed water was repelled from the polymer network, leading to increased cross-linking density. As a result, the hydrogel became more rigid and had reduced flexibility and increased modulus value.

### 3.8. Mechanical Properties

**Figure 7:**
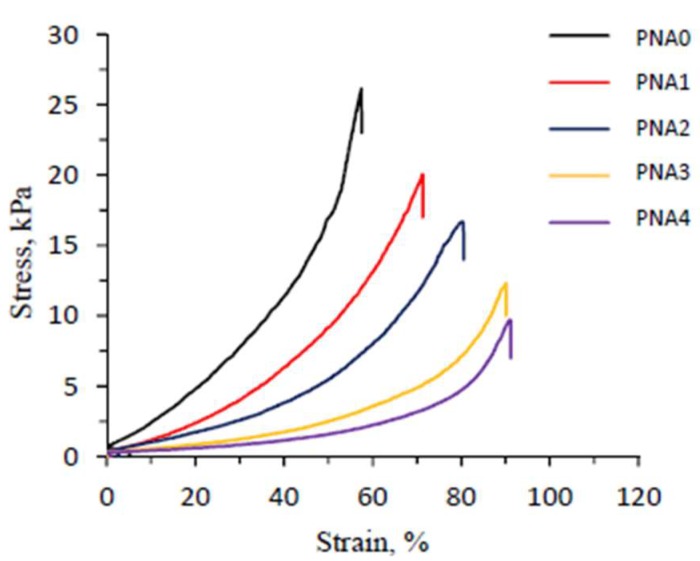
Stress-strain curves of conventional pNIPAM and semi-IPN hydrogels at 25 °C.

The stress-strain curves of conventional pNIPAM and semi-IPN hydrogels at room temperature are shown in Figure 7. The compressive modulus, fracture strain, and fracture stress of the hydrogels are listed in Table 3. It shows that compressive modulus and fracture stress decreased with pNIPAM content in the semi-IPN hydrogel, and the conventional pNIPAM hydrogel had the highest values. These results confirm that semi-IPN hydrogels with higher pNIPAM content have higher cross-linking density, which helps increase the modulus and the ability to resist stress. In addition, pore size also had an important effect on the mechanical strength of the semi-IPN hydrogels. Figure 5 and Table 3 confirm that the PNA4 sample had the largest pore size and least mechanical strength. This was because the hydrogels with small pore size prevented crack propagation, which was subjected to external forces because of the low stress concentration [45]. In other words, the stress distribution took place faster and easier in the hydrogel networks with smaller pore size.

Although it had the highest values of compressive modulus and fracture stress, the conventional pNIPAM hydrogel showed the lowest fracture strain, 57.5 ± 2.9%. Meanwhile, the semi-IPN hydrogels had a fracture strain of 71.2 ± 3.5 and 91.1 ± 4.9% for PNA1 and PNA4 samples, respectively. The results demonstrate that linear copolymer p(NIPAM-co-IAM) increases the fracture strain of semi-IPN hydrogels. The effect of the linear copolymer on fracture strain can be explained by its high viscosity, which helps energy dissipation take place rapidly during deformation. In addition, the semi-IPN hydrogels were highly hydrophilic, which helped the water to be more stable in the network, leading to significantly increased fracture strain, because water acted as a plasticizer [46]. Similar results can also be found in the literature, such as the poly[(*N*-isopropylacrylamide)-co-(aminoethyl methacrylate β-cyclodextrin)]/poly(*N*-isopropylacrylamide) system [17], salecan/poly(*N*-isopropylacrylmaide) system [27], and salecan/poly(*N*,*N*-dimethylacrylamide-co-2-hydroxyethyl methacrylate) system [47]. The above results indicate that the semi-IPN hydrogel had better mechanical properties than the conventional pNIPAM hydrogel.

### 3.9. Swelling Behavior

#### 3.9.1. LCST Behavior

**Figure 8:**
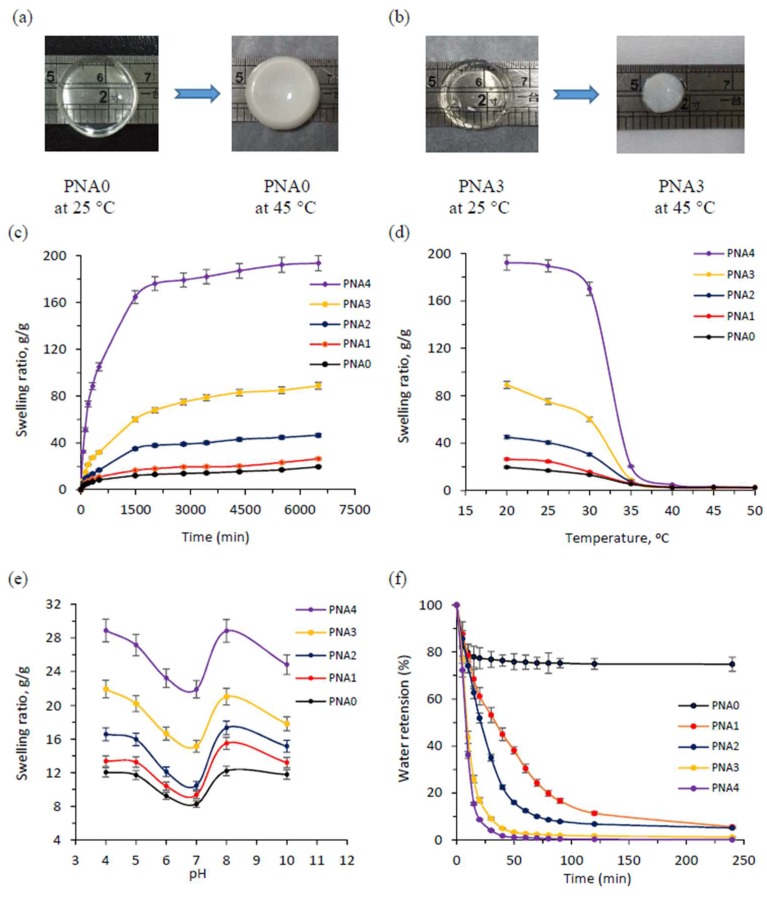
Photographs of (**a**) conventional pNIPAM and (**b**) semi-IPN hydrogels in the swollen state at 25 °C and the shrunken state at 45 °C in deionized water; (**c**) swelling kinetic curves in deionized water at 20 °C; (**d**) swelling ratio values in deionized water as a function of temperature from 20–50 °C; (**e**) swelling ratio values in PBS with pH values ranging from 4 to 10 at 25 °C for conventional pNIPAM and semi-IPN hydrogels; and (**f**) deswelling kinetic curves of hydrogels as temperature changed from 25 to 45 °C in deionized water.

Figure 8a,b show images of PNA0 and PNA3 samples in the swollen state at 25 °C (below LCST) and the shrunken state at 45 °C (above LCST). At the beginning, both samples were highly transparent. After being immersed in DI water at 45 °C, the PNA3 sample turned opaque, while the PNA0 sample maintained a transparent state. This phenomenon confirmed that the semi-IPN hydrogel was more thermo-responsive than the conventional pNIPAM hydrogel. The change in the diameter of the hydrogel samples was also measured after immersing them in DI water at 45 °C for 12 h. For the PNA0 sample, the diameter changed from 16 mm to 15 mm, while the PNA3 sample showed a stronger collapse from 16 mm to 8 mm. The results can be explained by the heat conduction and thermal sensitivity of linear copolymer p(NIPAM-co-IAM). As noted above, the conventional pNIPAM hydrogel possessed only a three-dimensional network with densely cross-linking density, while the semi-IPN hydrogel had linear copolymer chains entangled within the pNIPAM network. The linear copolymer acted as a thermal conductor, which helped the heat transfer in the semi-IPN hydrogels take place faster than in the conventional pNIPAM hydrogel. Moreover, at 25 °C the linear copolymer was in the relaxed state, leading to swelling of semi-IPN hydrogels. When the temperature was raised to 45 °C, the linear copolymers moved through the coil-to-globule transition [36]. Therefore, the polymer network of semi-IPN hydrogel was pushed closer together, leading to stronger collapse and larger deformation compared to the conventional pNIPAM hydrogel. The transition is schematically illustrated in Figure 1c.

#### 3.9.2. Swelling Kinetics and Equilibrium Swelling Ratio

Figure 8c shows the swelling kinetic curves of the hydrogels in DI at 20 °C. It can be seen that the swelling rate and equilibrium swelling ratio of the hydrogels obviously increased from PNA0 to PNA4 samples. These results can be explained by the hydrophilicity of the linear copolymer p(NIPAM-co-IAM) introduced into the semi-IPN hydrogels. When the linear copolymer content increased, the semi-IPN hydrogels became more hydrophilic and absorbed more DI water, leading to an increased equilibrium swelling ratio. Moreover, it is also believed that the high-molecular-weight chains in the semi-IPN hydrogel acted as channels for water molecules [29]. Thus, the linear copolymer could help water molecules travel easily from outside into the hydrogel network, leading to an increased swelling rate. It has also been reported that swelling kinetics are influenced by the cross-linking density of the polymer networks [48]. In this work, the amounts of NIPAM monomer and MBA cross-linker were decreased from PNA0 to PNA4 samples, causing lower cross-linking density. In addition to high hydrophilicity, the free space inside the hydrogels may be an important factor influencing the swelling rate and equilibrium swelling ratio. Thus, the PNA4 sample had the largest pore size, highest swelling rate, and highest swelling ratio. This result further demonstrates that the swelling kinetics of semi-IPN hydrogels can be controlled by modifying their linear copolymer content.

#### 3.9.3. Temperature Dependence

The thermo-sensitive property of the semi-IPN hydrogels is demonstrated in Figure 8d. In the temperature range of 20 to 30 °C, all of the hydrogel samples had high swelling ratios and a negligible decreasing trend. In the temperature range of 30 to 35 °C, there was a significant decreasing trend in the swelling ratios. Such a phenomenon could be explained by the strong influence of temperature on the phase transition of semi-IPN hydrogels. It can be noted from Figure 4b that the LCST values of the hydrogels varied from 31.6 to 32.7 °C for PNA0 and PNA4 samples, respectively. Generally, the thermo-responsive semi-IPN hydrogel shrunk above the LCST and swelled below the LCST. The LCST of the thermo-responsive hydrogel could be influenced by introducing hydrophobic or hydrophilic co-monomers to its network. In this work, the IAM hydrophilic monomer was introduced into the semi-IPN hydrogels, leading to gradually increased LCSTs and swelling ratios of the obtained hydrogels. However, Figure 8d shows no significant difference in the swelling ratios of the hydrogels at temperatures above LCST. This suggests that semi-IPN samples retracted to form the same network structure at these temperatures.

#### 3.9.4. pH Dependence

Besides being thermo-responsive, the semi-IPN hydrogels also showed pH-responsive properties. Figure 8e shows the swelling ratios of the hydrogels in pH buffer solution ranging from pH 4 to 10 at 25 °C. The swelling ratios of the semi-IPN hydrogels were higher than those of the conventional pNIPAM hydrogel. For the semi-IPN hydrogels, the swelling ratios increased as the linear copolymer p(NIPAM-co-IAM) content increased. This may be due to the influence of the high hydrophilic IAM monomer, as discussed above. In addition, Figure 8e shows that the swelling ratio of each hydrogel was high at pH 4, gradually decreased as pH value increased, and reached the lowest value at pH 7. Then it raised sharply again at pH 8 but decreased a little at pH 10.

**Figure 9:**
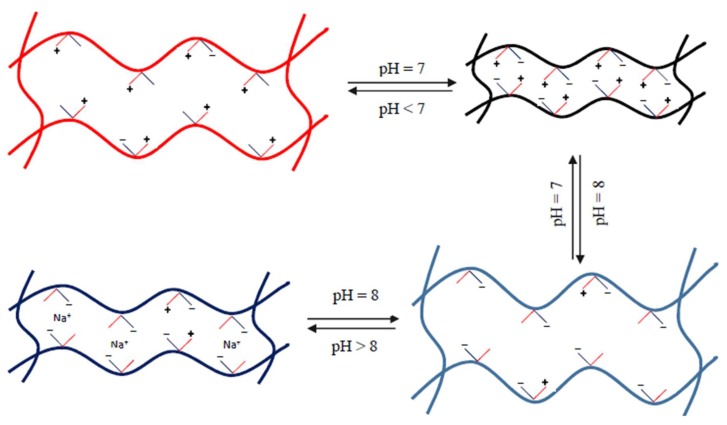
Mechanism of the influence of pH value on the swelling ratio of semi-IPN hydrogel.

It was reported that the swelling ratios of hydrogel are influenced by the isoelectric point and the repulsion of moieties with the same type of charge in the material system. First of all, it is necessary to assume that in the aqueous solution, the functional groups –COOH and –NH_2_ are readily ionized to –COO^−^ and NH_3_^+^. In the pH 7 buffer solution, the negatively and positively charged moieties were probably balanced and the hydrogel system reached the isoelectric point. At this point, the repulsion of moieties with the same type of charge almost disappeared, while the attraction of –COO^–^ and NH_3_^+^ became strong and shrinkage took place in the hydrogel network, leading to the lowest swelling ratio. In the acidic buffer solutions (pH 4, pH 5, and pH 6), the functional groups –NH_2_ were ionized into –NH_3_^+^, leading to a greater quantity of positive charges than negative charges. On the contrary, in the pH 8 buffer solution, the –COOH groups were ionized into –COO^–^, resulting in a greater quantity of negative charges than positive charges. In both cases, the hydrogel networks expanded rapidly by electrostatic repulsion, leading to increased swelling ratios. However, as the pH value went up from 8 to 10, the swelling ratios reduced slightly. This may be because the existence of alkaline ions in the swelling environment with high concentration caused a “charge screening effect” [49]. These positive ions function to reduce the electrostatic repulsion between the same charged –COO^–^ groups. This caused the polymer structure to come closer and reduced expansion, leading to decreased swelling ratios. The mechanism for the influence of pH values on the swelling ratios of the hydrogels is illustrated in Figure 9.

#### 3.9.5. Deswelling Kinetics

Figure 8f shows the deswelling kinetics of the hydrogels. This was measured by transferring the equilibrium swollen hydrogels in DI water as the temperature changed from 25 °C to 45 °C. As mentioned above, when the temperature was higher than LCST, the hydrogel networks collapsed, since the absorbed water moved out. In Figure 8f, the semi-IPN samples show a faster shrinking rate compared to conventional pNIPAM hydrogel. Moreover, most of the water inside the semi-IPN hydrogels was removed when their structure collapsed, while the conventional pNIPAM hydrogel was only 25%. The water retention of semi-IPN hydrogels was influenced by the linear copolymer p(NIPAM-co-IAM) content. For example, after being immersed in DI water at 45 °C for about 250 min, the water retention values were 4.95% and 0.08% for PNA1 and PNA4 samples, respectively. In general, the conventional pNIPAM hydrogel formed from dense walls, which may have prevented water molecules from running out when it was immersed in the higher LCST condition. For the semi-IPN hydrogels, the hydrophilic linear copolymer provided channels, and water molecules easily moved out when their structure collapsed.

## 4. Conclusions

In this work, a novel p(NIPAM-co-IAM)/pNIPAM semi-IPN hydrogel based on NIPAM and IAM monomers was prepared by free radical polymerization. By introducing the linear copolymer p(NIPAM-co-IAM) into a solution of NIPAM monomer and MBA cross-linker, pH and thermo dual-responsive semi-IPN hydrogels were obtained. First, GPC and DLS tests showed that the IAM co-monomer reduced the molecular weight and increased the hydrodynamic diameter of the linear copolymer. The chemical structure, confirmed by FTIR and ^1^H NMR, suggests that linear polymer chains were entangled in pNIPAM networks. In addition, TGA, rheological, and mechanical tests were measured, indicating thermal stability, viscoelastic behavior, and good mechanical properties of the semi-IPN hydrogel. SEM images showed a uniform porous structure of the hydrogels with pore size ranging from 119 ± 16 to 297 ± 61 µm. Moreover, the LCST and swelling properties of the hydrogels in DI water and buffer solution with pH ranging from 4 to 10 at different temperatures were measured. The LCST of the semi-IPN hydrogels in DI water increased slightly from 31.6 to 32.7 °C with increased linear copolymer content, while in pH buffer solution it was in the range of 25.2 to 34.7. The swelling ratios were influenced by linear copolymer content and pH environment: increased linear copolymer content or acid/base solution increased the swelling ratio. The swelling and deswelling rates of the semi-IPN hydrogels were also significantly accelerated with increased linear copolymer content. The phase transition and swelling behavior of the semi-IPN hydrogel in this study can be significantly changed by a change of temperature and/or pH environment, indicating that it can potentially be used for a drug delivery system or as an absorbing material.

## Figures and Tables

**Table 1:** Composition of raw materials used for the preparation of semi–IPN hydrogels.

Samples	Linear Copolymer p(NIPAM-co-IAM) Solution (mL)^a^	NIPAM (g)	DI Water (mL)	MBA (mg)	APS^b^ Solution (mL)	TEMED^c^ Solution (mL)	Total Volume (mL)
PNA0	0	1.00	3.1	40.0	2.3	4.6	10
PNA1	1	0.77	3.6	30.8	1.8	3.6	10
PNA2	2	0.69	3.2	27.6	1.6	3.2	10
PNA3	3	0.60	2.8	24.6	1.4	2.8	10
PNA4	4	0.52	2.4	20.0	1.2	2.4	10

^a^Solution of 1 mg/10 mg DI water; ^b^solution of 0.4792 g APS/25 mL DI water; ^c^solution of 4 mL TEMED/25 mL DI water.

**Table 2:** Molecular weight determination of linear copolymer p(NIPAM-co-IAM) and homopolymer pNIPAM. PDI, polydispersity index.

Sample	Mn.10^4^ (g/mol)	PDI
Linear copolymer p(NIPAM-co-IAM)	5.26	3.4
Linear homopolymer pNIPAM	23.9	3.6

**Table 3:** Pore sizes and compressive properties of conventional pNIPAM and semi-IPN hydrogels.

Sample	Pore Size (µm)	Compressive Modulus (kPa)	Fracture Strain (%)	Fracture Stress (kPa)
PNA0	112 ± 51	30.3 ± 3.1	57.5 ± 2.9	26.1 ± 2.7
PNA1	119 ± 16	23.1 ± 2.4	71.2 ± 3.5	20.2 ± 2.1
PNA2	161 ± 40	12.4 ± 1.1	80.4 ± 3.9	16.6 ± 1.5
PNA3	253 ± 43	5.2 ± 0.6	90.1 ± 4.7	12.3 ± 1.1
PNA4	297 ± 61	3.2 ± 0.4	91.1 ± 4.9	9.7 ± 0.8
